# Satellite Cells Contribution to Exercise Mediated Muscle Hypertrophy and Repair

**DOI:** 10.22074/cellj.2016.4714

**Published:** 2016-09-26

**Authors:** Behzad Bazgir, Rouhollah Fathi, Mojtaba Rezazadeh Valojerdi, Paul Mozdziak, Alireza Asgari

**Affiliations:** 1 Exercise Physiology Research Center, Baqiyatallah University of Medical Sciences, Tehran, Iran; 2 Department of Embryology, Reproductive Biomedicine Research Center, Royan Institute for Reproductive Biomedicine, ACECR, Tehran, Iran; 3 Department of Anatomy, Faculty of Medical Sciences, Tarbiat Modares University, Tehran, Iran; 4 Physiology Graduate Program, North Carolina State University, Raleigh, NC, USA; 5 Aerospace and Subaquatic Medicine Faculty, Aerospace Medicine Research Center, AJA Medical Sciences University, Tehran, Iran

**Keywords:** Skeletal Muscle Satellite Cells, Resistance Training, Exercise, Plasticity

## Abstract

Satellite cells (SCs) are the most abundant skeletal muscle stem cells. They are widely recognized for their contributions to maintenance of muscle mass, regeneration and hypertrophy during the human life span. These cells are good candidates for cell therapy due to their self-renewal capabilities and presence in an undifferentiated form. Presently, a significant gap exists between our knowledge of SCs behavior and their application as a means for human skeletal muscle tissue repair and regeneration. Both physiological and pathological stimuli potentially affect SCs activation, proliferation, and terminal differentiation the former category being the focus of this article. Activation of SCs occurs following exercise, post-training micro-injuries, and electrical stimulation. Exercise, as a potent and natural stimulus, is at the center of numerous studies on SC activation and relevant fields. According to research, different exercise modalities end with various effects. This review article attempts to picture the state of the art of the SCs life span and their engagement in muscle regeneration and hypertrophy in exercise.

## Introduction

The adult human body consists of approximately 600 muscles which form the largest body tissue, about 40-50% of body weight. Skeletal muscle mononuclear stem cells, called satellite cells (SCs), have been first identified more than half a century ago by Mauro (1). Since then, there is tremendous interest to investigate their application in muscle regeneration and repair due to their special self-renewal and multi-differentiation capabilities (2). Their name pertains to their location, residing between the skeletal myofiber’s basal lamina and the plasma membrane. SCs play an important role in afterbirth growth and regeneration of skeletal muscle. 

Muscle fiber hypertrophy is driven by the addition of SC nuclei to existing myofibers. The SC population also holds the nuclear reserve to enable muscle regeneration; however, they are usually mitotically quiescent (3). In occasions of regeneration and healing, SCs can activate to produce myoblasts and myonuclei through subsequent steps of activation, proliferation and differentiation (2). Once activated, SCs self-renew to provide a population of quiescent and undifferentiated progenitor cells to replenish the SC pool or undergo differentiation in the myogenesis pathway. 

Since SCs are not the only cells with stemness properties in skeletal muscles, their identity as SCs is recognized by the presence of specific molecular markers. 

### Molecular markers of satellite cells 

SCs can be recognized with electron microscopy according to their location between the basal lamina and sarcolemma; however, this technique is too costly and time-consuming. In recent years systemic and gene markers such as myogenic differentiation 1 (MyoD) and paired box-7 (PAX7) have been used for their identification. However these markers should be used with caution as they are not translated equally in different animals (4). In addition, different arrangements of SC populations have been reported for various muscle groups (5). Altogether, PAX7 is considered to be a generally agreed upon biomarker for quiescent SC recognition. The SC population may be entirely assessed by a combination of MyoD, myogenin and PAX7 staining (6). It seems reasonable to measure the above mentioned markers to determine the state of SCs in rodents (4). Since SCs are normally quiescent and are pushed activated in response to different physiological and pathological stimuli, it is therefore essential to identify the exact mechanisms and factors that affect SC activation and their temporal variation throughout their life span. In order to unravel the complexity of this subject, SCs heterogeneity in an individual, both in number and spatial distribution within various skeletal muscles, is a tremendous challenge in this field, which is not attended due to lack of empirical data. 

### Satellite cell activation

**Fig.1:**
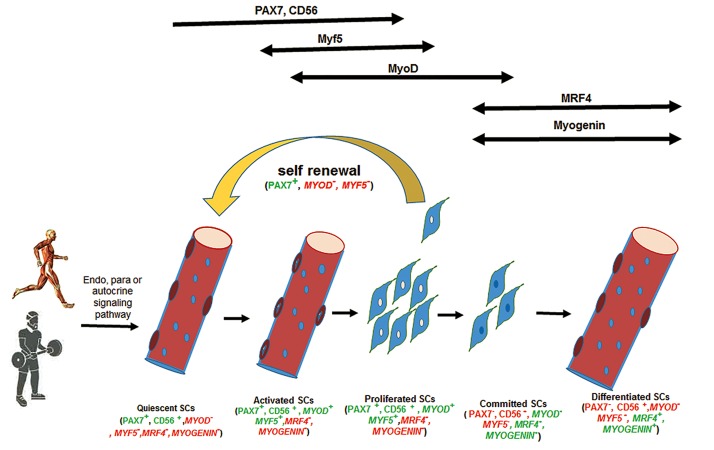
The morphological and functional changes of satellite cells (SCs) in response to exercise. Resistance and endurance exercise via endo-, para-, or autocrine mechanisms activate SC from a quiescent state where they undergo proliferation, commitment, and differentiation to add myonuclei to preexisting myofibrils or self-renew, and return to the quiescent state. During early stages of myogenic linage PAX7, CD56, and Myf5 derive the activation and proliferation process, however at later stages MRF4 and myogenin have a master regulatory role by controlling terminal differentiation (green color).

When a skeletal muscle is exercised or injured, at the cellular level SCs exit from their quiescent state and become activated. SCs subsequently proliferate, differentiate, and fuse into preexisting myofibers to create new myonuclei or return to the basal quiescence state (7). Little information exists about the molecular mechanism that underlies the aforementioned process. A family of proteins termed myogenic regulatory factors (MRFs) such as Myogenic factor -5 (Myf-5), MyoD, and myogenin control myogenic lineage progression. During the SCs activation process, the expression of MRFs increase and consequently these factors regulate myogenic progression (8). The involvement of MRFs, initiated by the first MRF (Myf-5), along with MyoD expression and proliferation increase in activated SCs (9). Subsequently and in the final step, observed increments in myogenin expression as a marker of the terminal differentiation occur (Fig.1) (10). 

Concomitant expressions of MyoD and PAX7 are well recognized as an SCs activation index (10,11), whereas in the absence of PAX7 and PAX3, Myf-5 is solely capable of myogenin activation resulting in myogenic differentiation. However, these SCs have no normal proliferation capacity and undergo apoptosis (11) which demonstrates the importance of PAX7 roles in normal SC behavior and skeletal muscle regeneration (12). In summary, SCs in response to different types of stimuli or under the control of MRFs can be activated, proliferated, differentiated, and ultimately participate in muscle regeneration. In the absence of MRFs, however, different steps in the SC life span can be blocked or inhibited. 

### Regulation of satellite cell activation

SCs are mitotically quiescent and their state is altered in response to a variety of physiologic and pathologic stimuli such as denervation (13), aging (14,16), sarcopenia (17), muscle burns (18), caloric restriction (19), and exercise (16,20,22). Physiological activators such as exercise (15), mechanical stretches (14,23), and aging (24,26), as well as pathologic conditions such as Duchenne muscular dystrophy (2,27) and irradiation (24,28) affect SC quiescence, their numbers, activation, and differentiation states through multiple signaling pathways. Regulatory factors of their behavior include elements present in their immediate microenvironment or the so-called niche that encompasses the structural elements, myofibers (29,31), local (autoand paracrine) factors secreted by interstitial cells, microvasculatures, neuromuscular junctions, immune cells (32), and systemic factors such as insulin-like growth factor-1 (IGF-I) and hepatocyte growth factor (HGF), the latter has recently been reported to play a triggering role in SC activation (33,35). Nitric oxide (NO) is also involved in SCs activation as mechanical stretching increases NO levels (14). 

### Satellite cell involvement in muscle hypertrophy

It is suggested that SC addition to skeletal muscle fibers positively promotes muscle hypertrophy (36). Muscle hypertrophy, an increase in the size of skeletal muscle fiber, which occurs in sports and strength training is a complex process affected through multiple sites and stimulatory/inhibitory factors such as neuromuscular junctions, endocrine molecules and myogenic regulatory genes, the net effect of all determines the gain in muscle mass. It has been reported that myonuclei have inhibitory "ceiling" or "restrictive" effects on myofiber hypertrophy (37). According to the nuclear domain theory, an increase in skeletal muscle myonuclei only occurs simultaneously with a substantial increment in the myofiber cross-sectional area (38,41). The nuclear domain theory states that nuclear complement occurs in the early juvenile nuclei growth through an increase in domain size (33). This theory implies that alterations in myonuclei number occur by at least two mechanisms: the addition of myonuclei through SCs during hypertrophy and a decline in myonuclei number in apoptosis and/or necrosis during atrophy of muscle fibers (41). SC-mediated myonuclear addition sustains muscle growth (37,42) when myonuclear domain peaks at ~ 2000 μm^2^ per nuclei (37,39,42). 

SC content and muscle mass decline with aging, however both old and young individual’s SCs appeared to have a similar response to exercise (43). Interestingly, this response was gender dependent, as an SC-induced hypertrophic response to resistance training (RT) was distinctly higher in male volunteers (44). Some studies revealed that an increase in SC content after RT only happened in young male volunteers (42,44). 

Myostatin is expressed in SCs and myoblasts, as one of the most potential inhibitory growth factors of muscle hypertrophy (33). Myostatin suppresses MyoD and increases SCs self-renewal capacity thus maintaining SCs in the quiescent state (27). Although myostatin inhibition induces muscle hypertrophy, this process seems to be SC-independent (45). Muscle hypertrophy and remodeling are complicated processes, hence it has been suggested that SCs play a pivotal role in this procedure. Its relative role and life-long importance in physiological states (i.e., gender, aging, endurance, and RT) and in pathologic conditions (i.e., Duchene muscular dystrophy and cachexia) need additional investigation. 

### Satellite cell involvement in skeletal muscle regeneration

The process of muscle regeneration consists of three phases: distraction, repair, and remodeling. Each step is controlled by multiple endo-, para-, and autocrine regulatory factors. However, SCs appear to be the main candidates that play an essential role in the muscle regeneration process, especially in the latter two phases of repair and remodeling (30). 

According to Collins et al. (46), quiescent mice SCs grafted to a mice model of dystrophic muscles served operationally both in producing functional SCs and contributing to muscle fiber regeneration. However, rodent and human skeletal muscles host SCs that possess different features. First, there exist various amounts of SCs in human and rodent skeletal muscles. Second, the muscle regeneration capacity of mouse SCs seems independent from their niche. Quiescent isolated mouse SCs are capable of taking part in muscle regeneration to functionally increase the SCs pool of dystrophic muscles in mice (4,46). However, due to ethical considerations and difficulties in obtaining sufficient SCs for grafts and limitations in tracing transplanted SCs, an evidenced-based comparison is practically impossible and remains a highly challenging technique for future human randomized controlled trials. Retrieval of SCs from human skeletal muscles is currently impractical for as soon as the SCs are isolated, they face a new *in vitro* niche where they are suddenly exposed to high oxygen. This particular environment suites their path towards differentiation (47). It is therefore a necessity to comprehensively identify the life span of human SCs and recognize modulatory factors that affect their state and fate. 

Negative and positive activators of SCs include macrophages (48,49), microvasculature (50), neuromuscular junctions (51), smooth muscle cells, fibroblasts (52), and muscle fibers (29,31). Various activators can affect the SC life cycle and their capacity to participate in muscle regeneration. A substantial bulk of evidence indicates that growth factors such as IGF-1 (53), leukemia inhibitory factor (LIF) (54), HGF and endogenous fibroblast growth factor (FGF) (55) increase myoblast induced muscle regeneration in humans and mice (56). However, growth factor isoforms have different effects. In contrast to IGF-1 (16), IGF-6 has either no (18) effect or a suppressing effect (20) on SC induced muscle regeneration. 

Our current understanding about changes in SCs differentiation within a skeletal muscle is poor, especially with exercise influences. Carlson (57) have reported that newly formed myofibrils can readily be distinguished by their small calibers and centrally located myonuclei in the early stages of muscle differentiation. However, they observed that late stages of muscle differentiation were morphologically and functionally indistinguishable from other myofibrils. Altogether, alterations in structural elements of a niche (i.e., basal lamina and myofibril), local and systemically secreted factors could affect SC activation, proliferation, terminal differentiation, and their involvement in muscle regeneration. 

### Exercise and satellite cell activation

Since the discovery of SCs in 1961 by Mauro (1), we have increased our understanding of the mechanisms of SCs activation and their final fate. Solid evidence shows that SCs decrease with increased age and during pathologic conditions such as muscle dystrophy (58). It has clearly been demonstrated that increased muscle activity augments SC proliferation (15) whereas muscle inactivity decreases SCs proliferation (22). 

Exercise and training can easily be undertaken and its parameters manipulated, hence this can be a natural, non-pharmacologic, promising route of intervention in regenerative medicine. Although exercise, as a physiologic stressor, could potentially exert modulatory effects during different stages of the SC life cycle, inconsistent reports exist on responses to different modes of exercise. Different exercise parameters (i.e., frequency, intensity, duration, and type/mode of exercise) should be taken into consideration when evaluating exercise-induced SC changes. As seen in Table 1, different exercise interventions can modulate SC activation in various ways (12). However, determining the exact volume, duration, and threshold of exercise training for optimal activation of SC in subjects at different physical fitness levels, ages, and gender is currently extremely difficult, if possible. 

### Satellite cell response to endurance exercise 

SCs activate from their quiescent state in response to events, such as micro-injuries that follow exercise. Inconsistencies and variations in SC content and activity after endurance training as reported in the literature can be attributed to differences in intensity, duration, and frequency of the administered exercise and/or to some extent to incompatible muscles studied. Time and intensity of exercise are important contributing factors in SCs activation. An increase in SC content has been reported after 40 to 155 minutes of moderate to high intensity endurance exercises (21,59,60), whereas a similar change after 30 minutes of low intensity exercise was not observed (61). This finding has implied that the intensity of the exercise is an important factor in SC activation and supports SC engagement in muscle regeneration (12). Similarly, Kurosaka et al. (62) have confirmed that increased in SC content depended upon the intensity rather than the duration of exercise. 

On the other hand, various muscle groups and types react differently to exercise interventions. Smith et al. have stated that rat SC content did not increase following one week of running on a treadmill for 30 minutes per day (61). In contrast, Parise et al. (21) reported a significant increase in SC number and activation in mice two and four days after they ran on a treadmill. Of note, Smith et al. (61) studied the soleus whereas Parise et al. (21) studied the anterior tibialis muscle, the former known to be mainly a slow type muscle (63). Type I fibers have more SCs than type II fibers and hence a lesser adaptive potential (12). Finally, the type of muscle contraction can modulate SCs response to exercise. Mangan et al. (59) have reported increased numbers of SCs 72 hours after rats engaged in 90 minutes of -13.5˚ non-fatiguing eccentric running on a motorized treadmill. In a study by Eksteen (64), well-trained runners completed 10 sessions of a high intensity running protocol during a 4-week downhill (6˚) and uphill (+15˚) running period. They were the first to report an SC proliferation response in the vastus lateralis (VL) muscle marked by increased CD56 (138%) and M-cadherin (123%). In that study both uphill and downhill running significantly increased SC terminal differentiation as interpreted by a remarkable 56% (uphill) and 60% (downhill) increase in myogenin. 

A limited number of studies regarded the effects of long-term endurance training (ET) on SCs (Table 1). Some animal studies reported an increase in SCs number and activation following 6-13 weeks of moderate to high intensity ET in young (65,67) and old (61,67) rats. 

Recently Fry et al. (65) and Joanisse et al. (66) reported an increase in SC activation following high and low intensity ET, respectively. In one study Snijders et al. (67) demonstrated that six months of moderate intensity ET intervention (75% baseline VO_2_ max) insignificantly affected the SC pool in obese diabetic subjects. However, since participants were obese diabetic patients under drug therapy, their training consisted of a combination of walking, skiing, and bicycle ergometer intervention, which made it unreasonable to compare the results. 

### Satellite cell response to resistance exercise in humans

Short-term SC response to resistance exercise (RE) is frequently studied by applying eccentric RE (ECC-RE) on the superficial and accessible VL muscle with an isokinetic dynamometer in a training regime which induces relatively maximum muscle micro-injuries (68,73). A comparison of ECC-RE and electrical stimulation in 8 young men has shown that electrical stimulation resulted in a higher increase in myogenin 24 hours after exercise. However, the study showed similar CD56 and PAX7 increments at 24, 96, and 192 hours following electrical stimulation than maximum ECCRE (74). 

Exercise intensity is believed to be one of the most detrimental functional factors that modify SCs response to RE. There are ample findings in support of the effectiveness of moderate to high intensity RE, either alone (68,72,73,75,80) or combined with sport supplements (81,82) on SC activation. The minimal threshold of RE intensity for SCs activation remains unidentified. A few evidences have demonstrated that even low intensity RE could stimulate SCs activation (75). 

Recently, low intensity RE [20-30% one repetition maximum (1 RM)] with blood flow restriction (BFR) has gained increasing interest because of their ability to increase SCs activation (83,84). Wernbom et al. (84) observed an increase in SCs response to low intensity RE (30% 1 RM) both with and without BFR. However, as study subjects performed BFR+RE with one leg followed by RE alone with the opposite leg (a 10-minute lapse between two), the cross-transfer effects impact of this exercise protocol at the systemic level might have influenced and biased the results. 

Alongside intensity, there exist other decisive factors in training such as exercise volume. It can be asserted that as with other modalities of exercises, an undetermined threshold exists for volume of the RE mode beyond which RE intervention induces SCs activation. A few researches have observed SC activation due to 70 to 300 repetitions of RE (68,70,73,77,78,83,85,86) (Table 1). Paulsen et al. (85) demonstrated by florescent microscopy and multiple labeling that 70 repetitions of eccentric elbow flexion (one round) resulted in SC activation, proliferation, and consequent terminal differentiation (myoblast fusion). The acute SC response to short-term RE was observed to occur 24 hours following exercise according to O’Reily et al. (72) who failed to report CD56 positive SCs 4 hours following knee extensor muscle ECC-RE. Instead, they observed elevations in SC numbers of 147% (1 day), 138% (3 days), and 118% (5 days) after exercise. Similar results by Mackey et al. (87) confirmed that a 24-hour period was required for SC response to exercise. 

Crameri et al. (68) observed an increase in CD56 positive SC two days after a one-bout knee ECC muscle contraction (3 sets×70 repetitions), which persisted up to 8 days following the exercise. This was one of the longest follow-up studies registered. The authors reported a 146-192% increase in SC during the 2to 8-day exercise period in young men. However, shorter periods of time were not investigated due to limitations in sample size. The short-term response of SC to RE in young and old volunteers investigated by Dreyer et al. (78) showed an increase in SCs numbers, both in young (141%) and old (51%) subjects 24 hours after maximal knee extensor muscle ECCRE (92 repetitions). 

Taken together, it appears that one bout of moderate to high intensity RE upregulates SC activation markers (CD56, PAX7, MyoD) in young and adult subjects. It seems that a complete SC response to RE exercise takes place 72-96 hours following exercise bouts, where SC number peaks at its highest value (68,72,85,87,88). Most studies analyzed SC responses in young volunteers after ECC-RE session(s), yet have not thoroughly investigated other possible confounding factors such as gender and exercise attributes (intensity, frequency, and duration). These seem to be as promising research venues in the future (59,89,91). It is believed that long-term systematic exercise training interventions can induce distinctive and positive effects on both muscle structure and function. 

### Satellite cell response to resistance training

A number of published studies researched the effects of long-term RT on SCs. A few reported that 6-20 weeks of RT protocol per se (37,43,44,79,89,92,93) or along with sport supplements (94,100) increase SCs content and activation (Table 1). The duration of RT administered ranged from 30-64 sessions of moderate to high intensity RT (60-80% 1 RM). Bellamy et al. (95) investigated the effects of 16 weeks (64 sessions) whole body high intensity RT (75-80% 1 RM) with whey protein on SC behaviors. They reported that their training regime induced a 47% increase in posttraining SCs content (95). 

### Satellite cell response to long-term resistance versus endurance training

Few comparative studies investigated SCs response to long-term RT and ET. Verney et al. (98) observed a similar increase in SC content in the deltoid and VL muscles after 14 weeks of resistance and ET in the same subjects. However, due to different muscle fiber type compositions, the rationale for a direct comparison was missing. Burd et al. (75) compared the effects of three RT intensities (90% 1 RM to failure, 30% 1 RM to failure, and 30% work load match with 90% 1 RM) on SC state and reported that PAX7^+^ SCs increased in all three groups, whereas MyoD and myogenin only enhanced in the 30% 1RM to failure group. The authors suggested that low intensity high volume RT was a more potent anabolic stimuli than high intensity low volume RT. These findings have suggested that an optimal training volume threshold exists. As such, fatigue is a significant stimulus for activation, proliferation, and terminal differentiation of SCs. Of note, simultaneous RT and ET increases SC numbers with subsequent enhancement of muscle mass and myonuclei (37,43,99). However, endurance exercise could neither increase satellite cell content (100,101) nor myonuclei addition to preexisting host myofibers (37,42,102). 

SCs activation may induce improvement in the muscle regeneration process by SC self-renewal. 

At least to a certain extent, regulation of muscle mass growth is independent of exercise-induced satellite cell myonuclei donation. This concept is important in exercise prescription for elder patients who suffer from skeletal muscle weaknesses (i.e., aging sarcopenia and other pathologic conditions) where muscle mass growth and function have special priority. 

It appears that myofiber type-related differences exist in SC response to exercise. Although a few evidences demonstrate that types I and II myofibers have the same SC content (77,103), numerous studies reveal that type I fibers possess a greater resting SC number in rodents (26,102,104) and humans (26,93,102,104). A few investigations have compared responses of types I and II myofiber SCs to exercise. Some human studies showed no type-related differences in SC content in young subjects (24,25,67). In response to training, the number of type II myofiber SCs increased, yet type I showed no increase (93,102). This process seems reasonable as type II have a greater contribution to muscle mass (hypertrophy) and higher responsiveness to RT. However, this proposed assumption needs future clarification. 

The mechanisms of exercise induced SCs activation are not completely identified. However, the early view towards the effects of exercise on SCs activation is that exercise induces micro-damage, wear, and tear in skeletal muscle tissue, where compensatory inflammatory responses follow and lead to SCs activation. However, recent data have shown activation of SCs after a non-damaging low intensity exercise (75). 

Exercise, as a potent stimulus, can alter endo-, para-, and autocrine regulators of SCs behavior (Fig.1). It is well known that exercise increases systemic levels of SCs that control cytokines (87,105), endocrine hormones (106,107), and growth factors (107,108). Paracrine factors arise from microvasculature (109) and neuromuscular structures. Finally, different types of exercise (especially eccentric contractions) can impose mechanical strain and sarcolemma damage (110) as important mediators of niche and SCs activities. Alongside these mechanisms exercise may affect negative regulators of SCs behavior, including myostatin and transforming growth factor-beta (TGF-β) (107,111,112). Exercise is reported to halt myostatin inhibitory effects on SCs behaviors (113,114). However, detailed mechanisms of how exercise affects SCs behaviors need more research. 

SCs are activated even after short-term exercise, but increases in SC numbers only occur after longterm RT or ET (12). Further research is needed to clarify the exact distinctive and interactive role of exercise and SC regulatory factors in order to highlight mechanism(s) involved in skeletal muscle adaptation to exercise during different physiological and pathological (muscle dystrophy, cachexia, etc.) conditions. 

There are no documented human SC studies in Iran (115). However, a few studies have applied skeletal muscle stem cell for differentiation into adult cardiomyocytes (116,118). Sharifiaghdas et al. (119) extracted skeletal muscle stem cells derived from patients’ rectus abdominis muscles. They successfully produced multinuclear myotube in culture and employed them to treat senile dysfunctional external sphincter muscles. As a weak point of this study, the authors did not report the patients’ ages. Salesi et al. (120) documented the effect of 8 weeks of moderate endurance exercise (70-80% VO_2_ max) along with a estrogen supplement on SCs in ovariectomized rats. They have observed a 1.5-fold increase in SC numbers as in the 8-week exercise-only group, assessed by the CD56 marker, whereas estrogen supplement per se and when accompanied with exercise, resulted in 73.9 and 68% decrease in SC numbers, respectively. Muscle samples were taken from the soleus muscle, a predominantly slow muscle which possesses greater resting SC levels and is less responsive to training compared to fast muscles (17). It is therefore reasonable to assess different myofiber isoforms for better evaluation of SC response to different exercise modalities. 

Khadivi-Borujeny et al. (121) measured a few SC modulatory factors: myostatin, TGF-β, and FGF-2 after 8 weeks of low load RT (30% of body weight). They observed a significant decrease in myostatin, increased FGF-2, and no change in TGF-β. The authors did not directly measure the SC markers. Hence it is difficult to draw a conclusion about effectiveness of an RE regime in the SC life cycle. Moreover, they did not report the animals’ weights at the end of 8-week RE program. Assay limitations in measuring animal percent body fat was asserted as a weakness of the study according to the authors. 

A review article by Jamalpoor et al. (122) concluded that acquisition of theoretical and empirical skills to obtain SCs, activate, proliferate, and differentiate into adult myofibers and ultimately into 3-D skeletal muscle substitutes are amongst many research areas that may repair muscle loss and injuries. The use of SCs, not only in muscle repair and regeneration, but also in advancing athletes’ and soldiers’ skeletal muscle powers beyond current biological limitations is envisioned as a future goal. 

**Table 1:** The effects of various shortand long-term exercises/interventions on SC activation


Mode of intervention	Effects on SC activation	References

Resistanceexercise(RE)	ST	↑	(73,75-82,88,114,115)
	LT	↑	(32,38,39,92,95-97,116)
Endurance exercise	ST	↔↑	(59,117,118)
	LT	↔↑	(68-70,79,115,116)
Supplements and exercise	ST	↔↑	(84,85,118,119)
	LT	↑↔	(84,98-100,119)
Sprint exercise	ST	?	-
	LT	↑	(66)
Concurrent exercise	ST	↓↑	(98,123-125)
	LT	?	-
HIIT	ST	↑	(76,126)
	LT	?	-
BFRE		↑	(83,84)
Electrical stimulation		↑	(74)
Irradiation		↓	(28,39)
Drug and exercise		↓	(71)


ST; Short-term, LT; Long-term, SC; Satellite cell, HIIT; High intensity interval training, BFRE; Blood fow restricton exercise, ↓; Decrease, ↔; Unchanged, ↑; Increase, and ?; Undetermined.

## Conclusion

SCs are the most important skeletal muscle resident stem cell that are known to reduce in numbers with aging, sarcopenia, and diseases such as muscle dystrophy. In contrast, physiological stimuli such as RT and ET and muscle micro-injuries that follow will initiate positive compensatory adaptations either by an increase in SC number or by suppression of the aging-dependent reduction in SC content. Exercise mode, volume, intensity and duration is to be optimized in order to activate, proliferate, and differentiate SCs. These are considered hot topics in the stem cell and exercise research arena. Some studies suggest that exercise intensity is the most important factor that determines SC activation, whereas others claim it is the volume that plays an essential role. Taken together, a determining optimum threshold of various exercise protocols for SCs stimulation is a research venue that needs further elucidation. Comprehensive, multicenter, inter-racial studies are necessary; to focus on SC modulator factors (e.g. age, gender and fitness levels of participants) where data comparison make human interventions possible. 

## References

[1] Mauro A (1961). Satellite cell of skeletal muscle fibers. J Biophys Biochem Cytol.

[2] Morgan JE, Zammit PS (2010). Direct effects of the pathogenic mutation on satellite cell function in muscular dystrophy. Exp Cell Res.

[3] Schultz E, Gibson MC, Champion T (1978). Satellite cells are mitotically quiescent in mature mouse muscle: an EM and radioautographic study. J Exp Zool.

[4] Boldrin L, Muntoni F, Morgan JE (2010). Are human and mouse satellite cells really the same?. J Histochem Cytochem.

[5] Ono Y, Boldrin L, Knopp P, Morgan JE, Zammit PS (2009). Muscle satellite cells are a functionally heterogeneous population in both somite-derived and branchiomeric muscles. Dev Biol.

[6] Schultz E, Chamberlain C, McCormick KM, Mozdziak PE (2006). Satellite cells express distinct patterns of myogenic proteins in immature skeletal muscle. Dev Dyn.

[7] Dhawan J, Rando TA (2005). Stem cells in postnatal myogenesis: molecular mechanisms of satellite cell quiescence, activation and replenishment. Trends Cell Biol.

[8] Sabourin LA, Rudnicki MA (2000). The molecular regulation of myogenesis. Clin Genet.

[9] Zammit PS, Heslop L, Hudon V, Rosenblatt JD, Tajbakhsh S, Buckingham ME (2002). Kinetics of myoblast proliferation show that resident satellite cells are competent to fully regenerate skeletal muscle fibers. Exp Cell Res.

[10] Cornelison DDW, Wold BJ (1997). Single-cell analysis of regulatory gene expression in quiescent and activated mouse skeletal muscle satellite cells. Dev Biol.

[11] Relaix F, Montarras D, Zaffran S, Gayraud-Morel B, Rocancourt D, Tajbakhsh S (2006). Pax3 and Pax7 have distinct and overlapping functions in adult muscle progenitor cells. J Cell Biol.

[12] Martin NRW, Lewis MP (2012). Satellite cell activation and number following acute and chronic exercise: a mini review. Cell Mol Exerc Physiol.

[13] Wozniak AC, Kong J, Bock E, Pilipowicz O, Anderson JE (2005). Signaling satellite cell activation in skeletal muscle: markers, models, stretch, and potential alternate pathways. Muscle Nerve.

[14] Wozniak AC, Pilipowicz O, Yablonka-Reuveni Z, Greenway S, Craven S, Scott E (2003). C-Met expression and mechanical activation of satellite cells on cultured muscle fibers. J Histochem Cytochem.

[15] Darr KC, Schultz E (1987). Exercise-induced satellite cell activation in growing and mature skeletal muscle. J Appl Physiol.

[16] Neuhaus P, Oustanina S, Loch T, Krüger M, Bober E, Dono R (2003). Reduced mobility of fibroblast growth factor (FGF)-deficient myoblasts might contribute to dystrophic changes in the musculature of FGF2/FGF6/mdx triplemutant mice. Mol Biol Cell.

[17] Bankol LC, Feasson L, Ponsot E, Kadi F (2013). Fibre type specific satellite cell content in two models of muscle diseases. Histopathology.

[18] Fiore F, Sébille A, Birnbaum D (2000). Skeletal muscle regeneration is not impaired in mutant mice. Biochem Biophys Res Commun.

[19] Cerletti M, Jang YC, Finley LW, Haigis MC, Wagers AJ (2012). Short-term calorie restriction enhances skeletal muscle stem cell function. Cell Stem Cell.

[20] Floss T, Arnold HH, Braun T (1997). A role for FGF-6 in skeletal muscle regeneration. Genes Dev.

[21] Parise G, McKinnell IW, Rudnicki MA (2008). Muscle satellite cell and atypical myogenic progenitor response following exercise. Muscle Nerve.

[22] Schultz E (1984). A quantitative study of satellite cells in regenerated soleus and extensor digitorum longus muscles. Anat Rec.

[23] Tatsumi R, Hattori A, Ikeuchi Y, Anderson JE, Allen RE (2002). Release of hepatocyte growth factor from mechanically stretched skeletal muscle satellite cells and role of pH and nitric oxide. Mol Biol Cell.

[24] Verdijk LB, Koopman R, Schaart G, Meijer K, Savelberg HHCM, van Loon LJC (2007). Satellite cell content is specifically reduced in type II skeletal muscle fibers in the elderly. Am J Physiol Endocrinol Metab.

[25] Verdijk LB, Dirks ML, Snijders T, Prompers JJ, Beelen M, Jonkers RAM (2012). Reduced satellite cell numbers with spinal cord injury and aging in humans. Med Sci Sports Exerc.

[26] Shefer G, Van de Mark DP, Richardson JB, YablonkaReuveni Z (2006). Satellite-cell pool size does matter: defining the myogenic potency of aging skeletal muscle. Dev Biol.

[27] Shi X, Garry DJ (2006). Muscle stem cells in development, regeneration, and disease. Genes Dev.

[28] Mozdziak PE, Schultz E, Cassens RG (1996). The effect of in vivo and in vitro irradiation (25 Gy) on the subsequent in vitro growth of satellite cells. Cell Tissue Res.

[29] Brack AS, Rando TA (2007). Intrinsic changes and extrinsic influences of myogenic stem cell function during aging. Stem Cell Rev.

[30] Brack AS, Conboy MJ, Roy S, Lee M, Kuo CJ, Keller C (2007). Increased Wnt signaling during aging alters muscle stem cell fate and increases fibrosis. Science.

[31] Conboy MJ, Karasov AO, Rando TA (2007). High incidence of non-random template strand segregation and asymmetric fate determination in dividing stem cells and their progeny. PLoS Biol.

[32] Gopinath SD, Rando TA (2008). Stem cell review series: aging of the skeletal muscle stem cell niche. Aging Cell.

[33] Ten Broek RW, Grefte S, Von den Hoff JW (2010). Regulatory factors and cell populations involved in skeletal muscle regeneration. J Cell Physiol.

[34] Tatsumi R, Anderson JE, Nevoret CJ, Halevy O, Allen RE (1998). HGF/SF is present in normal adult skeletal muscle and is capable of activating satellite cells. Dev Biol.

[35] Anderson J, Pilipowicz O (2002). Activation of muscle satellite cells in single-fiber cultures. Nitric Oxide.

[36] Phillips SM (2014). A brief review of critical processes in exercise-induced muscular hypertrophy. Sports Med.

[37] Petrella JK, Kim JS, Mayhew DL, Cross JM, Bamman MM (2008). Potent myofiber hypertrophy during resistance training in humans is associated with satellite cell-mediated myonuclear addition: a cluster analysis. J Appl Physiol.

[38] Mozdziak PE, Schultz E, Cassens RG (1994). Satellite cell mitotic activity in posthatch turkey skeletal muscle growth. Poult Sci.

[39] Mozdziak PE, Schultz E, Cassens RG (1997). Myonuclear accretion is a major determinant of avian skeletal muscle growth. Am J Physiol.

[40] Mozdziak PE, Pulvermacher PM, Schultz E (2000). Unloading of juvenile muscle results in a reduced muscle size 9 wk after reloading. J Appl Physiol.

[41] Teixeira CE, Duarte JA (2011). Myonuclear domain in skeletal muscle fibers.A critical review. Arch Exerc Health Dis.

[42] Petrella JK, Kim JS, Cross JM, Kosek DJ, Bamman MM (2006). Efficacy of myonuclear addition may explain differential myofiber growth among resistance-trained young and older men and women. Am J Physiol Endocrinol Metab.

[43] Kadi F, Thornell LE (2000). Concomitant increases in myonuclear and satellite cell content in female trapezius muscle following strength training. Histochem Cell Biol.

[44] Kosek DJ, Kim JS, Petrella JK, Cross JM, Bamman MM (2006). Efficacy of 3 days/wk resistance training on myofiber hypertrophy and myogenic mechanisms in young vs.older adults. J Appl Physiol.

[45] Amthor H, Otto A, Vulin A, Rochat A, Dumonceaux J, Garcia L (2009). Muscle hypertrophy driven by myostatin blockade does not require stem/precursor-cell activity. Proc Natl Acad Sci USA.

[46] Collins CA, Gnocchi VF, White RB, Boldrin L, Perez-Ruiz A, Relaix F (2009). Integrated functions of Pax3 and Pax7 in the regulation of proliferation, cell size and myogenic differentiation. PLoS One.

[47] Di Mario JX, Stockdale FE (1995). Differences in the developmental fate of cultured and noncultured myoblasts when transplanted into embryonic limbs. Exp Cell Res.

[48] Malerba A, Vitiello L, Segat D, Dazzo E, Frigo M, Scambi I (2009). Selection of multipotent cells and enhanced muscle reconstruction by myogenic macrophage-secreted factors. Exp Cell Res.

[49] Chazaud B, Sonnet C, Lafuste P, Bassez G, Rimaniol AC, Poron F (2003). Satellite cells attract monocytes and use macrophages as a support to escape apoptosis and enhance muscle growth. J Cell Biol.

[50] Rhoads RP, Johnson RM, Rathbone CR, Liu X, TemmGrove C, Sheehan SM (2009). Satellite cell-mediated angiogenesis in vitro coincides with a functional hypoxiainducible factor pathway. Am J Physiol Cell Physiol.

[51] Tatsumi R, Wuollet AL, Tabata K, Nishimura S, Tabata S, Mizunoya W (2009). A role for calcium-calmodulin in regulating nitric oxide production during skeletal muscle satellite cell activation. Am J Physiol Cell Physiol.

[52] Abou-Khalil R, Le Grand F, Pallafacchina G, Valable S, Authier FJ, Rudnicki MA (2009). Autocrine and paracrine angiopoietin 1/Tie-2 signaling promotes muscle satellite cell self-renewal. Cell Stem Cell.

[53] Mourkioti F, Rosenthal N (2005). IGF-1, inflammation and stem cells: interactions during muscle regeneration. Trends Immunol.

[54] Kurek JB, Bower JJ, Romanella M, Koentgen F, Murphy M, Austin L (1997). The role of leukemia inhibitory factor in skeletal muscle regeneration. Muscle Nerve.

[55] Miller KJ, Thaloor D, Matteson S, Pavlath GK (2000). Hepatocyte growth factor affects satellite cell activation and differentiation in regenerating skeletal muscle. Am J Physiol Cell Physiol.

[56] Brimah K, Ehrhardt J, Mouly V, Butler-Browne GS, Partridge TA, Morgan JE (2004). Human muscle precursor cell regeneration in the mouse host is enhanced by growth factors. Hum Gene Ther.

[57] Carlson B (2011). Principles of regenerative biology.

[58] Parise G (2014). Satellite cells: promoting adaptation over a lifetime. Acta Physiol (Oxf).

[59] Mangan G, Bombardier E, Mitchell AS, Quadrilatero J, Tiidus PM (2014). Oestrogen-dependent satellite cell activation and proliferation following a running exercise occurs via the PI3K signalling pathway and not IGF-1. Acta Physiol (Oxf).

[60] Van de Vyver M, Myburgh KH (2012). Cytokine and satellite cell responses to muscle damage: interpretation and possible confounding factors in human studies. J Muscle Res Cell Motil.

[61] Smith HK, Maxwell L, Rodgers CD, McKee NH, Plyley MJ (2001). Exercise-enhanced satellite cell proliferation and new myonuclear accretion in rat skeletal muscle. J Appl Physiol.

[62] Kurosaka M, Naito H, Ogura Y, Machida S, Katamoto S (2012). Satellite cell pool enhancement in rat plantaris muscle by endurance training depends on intensity rather than duration. Acta Physiol (Oxf).

[63] Augusto V, Padovani CR, Campos GER (2004). Skeletal muscle fiber types in C57BL6J mice. Braz J Morphol Sci.

[64] Eksteen GJ (2006). Satellite cell proliferation in response to a chronic laboratory-controlled uphill vs.downhill interval training intervention.Presented for the M.Sc., Stellenbosch.University of Stellenbosch.

[65] Fry CS, Noehren B, Mula J, Ubele MF, Westgate PM, Kern PA (2014). Fibre type specific satellite cell response to aerobic training in sedentary adults. J Physiol.

[66] Joanisse S, McKay BR, Nederveen JP, Scribbans TD, Gurd BJ, Gillen JB (2015). Satellite cell activity, without expansion, after nonhypertrophic stimuli. Am J Physiol Regul Integr Comp Physiol.

[67] Snijders T, Verdijk LB, Hansen D, Dendale P, van Loon LJ (2011). Continuous endurance-type exercise training does not modulate satellite cell content in obese type 2 diabetes patients. Muscle Nerve.

[68] Crameri RM, Langberg H, Magnusson P, Jensen CH, Schrder HD, Olesen JL (2004). Changes in satellite cells in human skeletal muscle after a single bout of high intensity exercise. J physiol.

[69] Gibala MJ, MacDougall JD, Tarnopolsky MA, Stauber WT, Elorriaga A (1995). Changes in human skeletal muscle ultrastructure and force production after acute resistance exercise. J Appl Physiol.

[70] Cermak NM, Snijders T, McKay BR, Parise G, Verdijk LB, Tarnopolsky MA (2013). Eccentric exercise increases satellite cell content in type II muscle fibers. Med Sci Sports Exerc.

[71] Mikkelsen UR, Langberg H, Helmark IC, Skovgaard D, Andersen LL, Kjaer M (2009). Local NSAID infusion inhibits satellite cell proliferation in human skeletal muscle after eccentric exercise. J Appl Physiol.

[72] O'Reilly C, McKay B, Phillips S, Tarnopolsky M, Parise G (2008). Hepatocyte growth factor (HGF) and the satellite cell response following muscle lengthening contractions in humans. Muscle Nerve.

[73] Hyldahl RD, Olson T, Welling T, Groscost L, Parcell AC (2014). Satellite cell activity is differentially affected by contraction mode in human muscle following a work-matched bout of exercise. Front Physiol.

[74] Crameri RM, Aagaard P, Qvortrup K, Langberg H, Olesen J, Kjaer M (2007). Myofibre damage in human skeletal muscle: effects of electrical stimulation versus voluntary contraction. J Physiol.

[75] Burd NA, West DW, Staples AW, Atherton PJ, Baker JM, Moore DR (2010). Low-load high volume resistance exercise stimulates muscle protein synthesis more than highload low volume resistance exercise in young men. PLoS One.

[76] Nederveen JP, Joanisse S, Séguin CM, Bell KE, Baker SK, Phillips SM (2015). The effect of exercise mode on the acute response of satellite cells in old men. Acta Physiol (Oxf).

[77] Walker DK, Fry CS, Drummond MJ, Dickinson JM, Timmerman KL, Gundermann DM (2012). PAX7+ satellite cells in young and older adults following resistance exercise. Muscle Nerve.

[78] Dreyer HC, Blanco CE, Sattler FR, Schroeder ET, Wiswell RA (2006). Satellite cell number in young and older men 24 hours after eccentric exercise. Muscle Nerve.

[79] Mackey AL, Holm L, Reitelseder S, Pedersen TG, Doessing S, Kadi F (2011). Myogenic response of human skeletal muscle to 12 weeks of resistance training at light loading intensity. Scand J Med Sci Sports.

[80] Snijders T, Verdijk LB, Smeets JS, McKay BR, Senden JM, Hartgens F (2014). The skeletal muscle satellite cell response to a single bout of resistance-type exercise is delayed with aging in men. Age (Dordr).

[81] Farup J, Rahbek SK, Riis S, Vendelbo MH, de Paoli F, Vissing K (2014). Influence of exercise contraction mode and protein supplementation on human skeletal muscle satellite cell content and muscle fiber growth. J Appl Physiol.

[82] Shelmadine B, Cooke M, Buford T, Hudson G, Redd L, Leutholtz B (2009). Effects of 28 days of resistance exercise and consuming a commercially available pre-workout supplement, NO-Shotgun, on body composition, muscle strength and mass, markers of satellite cell activation, and clinical safety markers in males. J Int Soc Sports Nutr.

[83] Nielsen JL, Aagaard P, Bech RD, Nygaard T, Hvid LG, Wernbom M (2012). Proliferation of myogenic stem cells in human skeletal muscle in response to low load resistance training with blood flow restriction. J physiol.

[84] Wernbom M, Apro W, Paulsen G, Nilsen TS, Blomstrand E, Raastad T (2013). Acute low-load resistance exercise with and without blood flow restriction increased protein signalling and number of satellite cells in human skeletal muscle. Eur J Appl Physiol.

[85] Paulsen G, Egner IM, Drange M, Langberg H, Benestad HB, Fjeld JG (2010). A COX inhibitor reduces muscle soreness, but does not influence recovery and adaptation after eccentric exercise. Scand J Med Sci Sports.

[86] McKay BR, Toth KG, Tarnopolsky MA, Parise G (2010). Satellite cell number and cell cycle kinetics in response to acute myotrauma in humans: immunohistochemistry versus flow cytometry. J physiol.

[87] Mackey AL, Kjaer M, Charifi N, Henriksson J, Bojsen Moller J, Holm L (2009). Assessment of satellite cell number and activity status in human skeletal muscle biopsies. Muscle Nerve.

[88] Roberts MD, Dalbo VJ, Kerksick CM (2011). Postexercise myogenic gene expression: are human findings lost during translation?. Exerc Sport Sci Rev.

[89] Kadi F, Schjerling P, Andersen LL, Charifi N, Madsen JrL, Christensen LR (2004). The effects of heavy resistance training and detraining on satellite cells in human skeletal muscles. J physiol.

[90] Roth SM, Martel GF, Ivey FM, Lemmer JT, Tracy BL, Metter EJ (2001). Skeletal muscle satellite cell characteristics in young and older men and women after heavy resistance strength training. J Gerontol A Biol Sci Med Sci.

[91] Charifi N, Kadi F, Fasson L, Denis C (2003). Effects of endurance training on satellite cell frequency in skeletal muscle of old men. Muscle Nerve.

[92] Verdijk LB, Snijders T, Drost M, Delhaas T, Kadi F, van Loon LJC (2014). Satellite cells in human skeletal muscle; from birth to old age. Age (Dordr).

[93] Verdijk LB, Jonkers RA, Gleeson BG, Beelen M, Meijer K, Savelberg HH (2009). Protein supplementation before and after exercise does not further augment skeletal muscle hypertrophy after resistance training in elderly men. Am J Clin Nutr.

[94] Hulmi JJ, Kovanen V, Lisko I, Selänne H, Mero AA (2008). The effects of whey protein on myostatin and cell cycle-related gene expression responses to a single heavy resistance exercise bout in trained older men. Eur J Appl Physiol.

[95] Bellamy LM (2012). Temporal pattern of type II fibre-specific satellite cell expansion to exercise correlates with human muscle hypertrophy: potential role for myostatin.Presented for the Ph.D..

[96] Olsen S, Aagaard P, Kadi F, Tufekovic G, Verney J, Olesen JL (2006). Creatine supplementation augments the increase in satellite cell and myonuclei number in human skeletal muscle induced by strength training. J physiol.

[97] Verdijk LB, Gleeson BG, Jonkers RAM, Meijer K, Savelberg HHCM, Dendale P (2009). Skeletal muscle hypertrophy following resistance training is accompanied by a fiber type specific increase in satellite cell content in elderly men. J Gerontol A Biol Sci Med Sci.

[98] Verney J, Kadi F, Charifi N, Fasson L, Saafi MA, Castells J (2008). Effects of combined lower body endurance and upper body resistance training on the satellite cell pool in elderly subjects. Muscle Nerve.

[99] Bruusgaard JC, Johansen IB, Egner IM, Rana ZA, Gundersen K (2010). Myonuclei acquired by overload exercise precede hypertrophy and are not lost on detraining. Proc Natl Acad Sci.

[100] Hoppeler H, Howald H, Conley K, Lindstedt SL, Claassen H, Vock P (1985). Endurance training in humans: aerobic capacity and structure of skeletal muscle. J Appl Physiol.

[101] Ingjer F (1979). Effects of endurance training on muscle fibre ATP-ase activity, capillary supply and mitochondrial content in man. J physiol.

[102] Smith HK, Merry TL (2012). Voluntary resistance wheel exercise during post natal growth in rats enhances skeletal muscle satellite cell and myonuclear content at adulthood. Acta Physiol (Oxf).

[103] Kadi F, Eriksson A, Holmner S, Thornell LE (1999). Effects of anabolic steroids on the muscle cells of strength-trained athletes. Med Sci Sports Exerc.

[104] Shefer G, Rauner G, Yablonka-Reuveni Z, Benayahu D (2010). Reduced satellite cell numbers and myogenic capacity in aging can be alleviated by endurance exercise. PLoS One.

[105] Bazgir B, Salesi M, Koushki M, Amirghofran Z (2015). Effects of eccentric and concentric emphasized resistance exercise on IL-15 serum levels and its relation to inflammatory markers in athletes and non-athletes. Asian J Sports Med.

[106] Heinemeier K, Mackey A, Doessing S, Hansen M, Bayer M, Nielsen R (2012). GH/IGF-I axis and matrix adaptation of the musculotendinous tissue to exercise in humans. Scand J Med Sci Sports.

[107] Blaauw B, Reggiani C (2014). The role of satellite cells in muscle hypertrophy. J Muscle Res Cell Motil.

[108] McKay BR, O'Reilly CE, Phillips SM, Tarnopolsky MA, Parise G (2008). Co-expression of IGF-1 family members with myogenic regulatory factors following acute damaging muscle-lengthening contractions in humans. J physiol.

[109] Christov C, Chretien F, Abou-Khalil R, Bassez G, Vallet G, Authier FJ (2007). Muscle satellite cells and endothelial cells: close neighbors and privileged partners. Mol Biol Cell.

[110] Vijayan K, Thompson JL, Norenberg KM, Fitts R, Riley DA (2001). Fiber-type susceptibility to eccentric contractioninduced damage of hindlimb-unloaded rat AL muscles. J App Physiol.

[111] Langley B, Thomas M, Bishop A, Sharma M, Gilmour S, Kambadur R (2002). Myostatin inhibits myoblast differentiation by down-regulating MyoD expression. J Biol Chem.

[112] Thomas M, Langley B, Berry C, Sharma M, Kirk S, Bass J (2000). Myostatin, a negative regulator of muscle growth, functions by inhibiting myoblast proliferation. J Biol Chem.

[113] Raue U, Slivka D, Jemiolo B, Hollon C, Trappe S (2006). Myogenic gene expression at rest and after a bout of resistance exercise in young (18-30 yr) and old (80-89 yr) women. J Appl Physiol.

[114] MacKenzie MG, Hamilton DL, Pepin M, Patton A, Baar K (2013). Inhibition of myostatin signaling through Notch activation following acute resistance exercise. PLoS One.

[115] Bazgir B, Asgari A (2015). The interactive role of exercise and satellite cells in skeletal muscle regeneration and hypertrophy. The scientific and medical journal of Ebnesina.

[116] Baharvand H, Piryaei A, Rohani R, Taei A, Heidari MH, Hosseini A (2006). Ultrastructural comparison of developing mouse embryonic stem celland in vivo-derived cardiomyocytes. Mol Cell Biol.

[117] Hatami L, Valojerdi MR, Mowla SJ (2007). Effects of oxytocin on cardiomyocyte differentiation from mouse embryonic stem cells. Int J Cardiol.

[118] Taha MF VM (2008). Effect of bone morphogenetic protein-4 on cardiac differentiation from mouse embryonic stem cells in serum-free and low-serum media. Int J Cardiol.

[119] Sharifiaghdas F, Taheri M, Moghadasali R (2011). Isolation of human adult stem cells from muscle biopsy for future treatment of urinary incontinence. Urol J.

[120] Salesi M, Sheikhani Shahin H, Gramizadeh B, Tanideh N (2010). The effects of 8 week exercise and estrogen supllementation in overctomized rats. Sport Biosciences (Harakat).

[121] Khadivi Borujeny AR, Marandi M, Haghjooy Javanmard Sh, Rajabi H, Khadivi Burojeny Z, Khorshidi Behzadi M (2012). Effect of eight weeks of resistance training on some signaling factors affecting on the satellite cells in wistar rats. Journal of Isfahan Medical School.

[122] Jamalpoor Z, Asgari AR, Nourani MR (2012). Skeletal muscle tissue engineering: present and future. J Mil Med.

[123] Pugh JK, Faulkner SH, Jackson AP, King JA, Nimmo MA (2015). Acute molecular responses to concurrent resistance and high intensity interval exercise in untrained skeletal muscle. Physiol Rep.

[124] Snijders T, Verdijk LB, Beelen M, McKay BR, Parise G, Kadi F (2012). A single bout of exercise activates skeletal muscle satellite cells during subsequent overnight recovery. Exp Physiol.

[125] Babcock L, Escano M, D'Lugos A, Todd K, Murach K, Luden N (2012). Concurrent aerobic exercise interferes with the satellite cell response to acute resistance exercise. Am J Physiol Regul Integr Comp Physiol.

[126] Séguin C (2015). A comparison of differing modalities of exercise on the acute satellite cell responses in older adults.Presented for the Ph.D..

